# Dispositional orientation to the present and future and its role in pro-environmental behavior and sustainability

**DOI:** 10.1016/j.heliyon.2018.e00882

**Published:** 2018-10-26

**Authors:** Marc Wittmann, Anna Sircova

**Affiliations:** a Institute for Advanced Sustainability Studies, Potsdam, Germany; b Institute for Frontier Areas in Psychology and Mental Health, Freiburg, Germany; c Time Perspective Network, Copenhagen, Denmark

**Keywords:** Psychology, Economics, Ecology

## Abstract

With our attitudes and behavior, which aim at promoting sustainable behavior, we face a temporal dilemma – a temporal conflict between short-term and long-term interests. Accordingly, psychological time is an essential variable in understanding how people decide between options of short-term self-interest, which can be experienced at present, and long-term common interest, such as sustainable development with an outcome that lies far in the future. Present feelings are often so powerful that considerations of future events are neglected. Individuals differ in their emphasis on present and future dimensions. A stronger future orientation and a mindful present orientation are positive predictors of sustainable behavior; hedonistic and impulsive present orientations are negative predictors. We discuss the concept of the balanced time perspective as the propensity to consciously switch among the time orientations of past, present, and future. Fitting with their overall psychological profile, individuals with a balanced time perspective might display a range of sustainable attitudes and behaviors.

## 1 Introduction

The classic social dilemma pro-environmental policy making faces can be described as the contrast between the interest of a utility-maximizing individual on the one hand and the common interest, a community, or even the whole planet on the other hand. Individual interests are sometimes fulfilled at the expense of the common interest. For example, the private interest of watering the garden or having an extended shower during an extreme drought conflicts with the public interest of water conservation. Violation of the common interest might happen as economic self-interest through overharvesting and depletion of a common natural resource. People also tend to behave hedonistically. Since acting pro-environmentally and in the general interest is often less pleasurable, it costs more, and it may be more time-consuming, personal comfort often prevails (Steg et al., 2014). Detrimental effects of our actions and the related responsibility are dispersed over all members of a society, i.e. millions of people. One individual act of, say, watering the garden during a drought that threatens the common drinking-water supply is practically imperceptible. Although each act may seem trivial, in sum such actions do great harm. That is the *contributor's dilemma*: when involving public goods, it is better when everyone contributes equally to saving water than if no one does; however, it is better for an individual not to contribute to water conservation if everyone else does. Then the individual receives greater benefits as a result of the contributions of others (Parfit, 2013, p. 303).

One goal of pro-environmental policy making among others is to promote sustainable and cooperative behavior. A combination of two dilemmas, temporal and inter-personal, can arise as the conflict encompasses the weighing of short-term self-interest against the long-term common interest (Milfont et al., 2012; Sircova et al., 2015; van Lange and Joireman, 2008). Accordingly, the perception of time is an essential variable in understanding how people decide between these options. Decisions depend on the temporal constraints, i.e. when the outcome of a choice can be expected. Driving comfortably in your car to your favorite restaurant may only take 20 minutes, while using public transportation may take an hour. Regarding potential benefits, ‘now’ or ‘soon’ are generally more attractive than ‘later’. In economic terms, the time until the beneficial outcome of a decision can be received is viewed as a cost and is weighed against the benefits of that outcome (Read, 2001). Analysis of consumer behavior shows that money and time are traded against each other, for example when deciding on fees and respective delivery times of purchased consumer goods (Hantula and Bryant, 2005).

Time is perceived differently from person to person and attitudes towards time vary between individuals: waiting time might seem unbearably long for one person, while it is hardly an issue for somebody else (Jokic et al., 2018). For example, impulsive individuals who are easily bored when no distraction is at hand are more likely to overestimate durations they have to wait through (Wittmann and Paulus, 2008, 2016). Accordingly, for impulsive individuals a given waiting time for a delayed reward is associated with too high a cost, which in turn leads to the selection of an alternative choice with a more immediate outcome, even when the delayed reward has a higher value. More broadly speaking and fitting the aforementioned temporal dilemma regarding pro-environmental behavior, impulsivity can be defined as a pattern of unplanned actions without regard for the potential negative consequences that might occur later (Sweitzer et al., 2008).

Individual differences in estimating time clearly show that personality has to be taken into account to understand temporal conflicts in decision-making. What is considered short-term and long-term and how people decide in relation to outcomes with different delays differs from person to person.

We start this overview with giving a comprehensive introduction to relevant research of how humans perceive time and how this influences every-day decision making (chapter 2). Then we highlight evidence of how social and personal life events can change an individual's time orientation (chapter 3). The further aim of this paper is then to assess and integrate the available literature on individual differences in subjective time and how it relates to issues of sustainability. In this context, we summarize research on individual differences regarding present and future orientation as predictors of pro-environmental attitudes and behaviors (chapter 4): several studies on time perspective and environmental engagement have been conducted over the last years showing that an individually stronger future perspective is especially predictive (for a meta-analysis, see Milfont et al., 2012). Very recent empirical advancements pertain to the two related concepts of mindfulness (chapter 4) and balanced time perspective (chapter 5). Being more mindful and more balanced in time orientations, the latter being the ability to voluntarily switch time orientations, are predictive factors for ‘green’ behavior. To sum up, our review of empirical and conceptual work is embedded in a general evaluation of how people typically perceive time and how this influences decision-making in the context of questions regarding pro-environmental attitudes and behavior.

In this overview we focus on the question of how individual assessments of the time dimension predict environmentally-friendly behavior. It is obvious that the focus on the time dimension can explain only a limited amount of behavioral variance. A summary of influencing internal (psychological) and external (social) factors “indicates that the question of what shapes pro-environmental behavior is such a complex one that it cannot be visualized through one single framework or diagram” (Kollmuss and Agyeman, 2002). Regarding internal factors, very general opinions (weltanschauung) such as the political ideology (free-market ideation vs. support for policy intervention) or cognitive capacities such as the ability to understand complex adaptive systems (systems thinking) essentially influence decision-making (Lezak and Thibodeau, 2016). Related to the systems thinking approach, the concept of ‘affinity towards diversity’, defined as valuing and liking biological and socio-cultural diversity has shown to correlate with ‘green’ attitudes and behavior (Corral-Verdugo et al., 2009). Our emphasis on the temporal dimension is not merely another internal factor adding to the existing list. As we will show, subjective time and the orientation in time play a pivotal role in decision-making. Everyday decisions are based on whether and how we emphasize the present and future and how we perceive and anticipate duration (Wittmann and Paulus, 2016).

## 2 Main text

### 2.1 “Warm” feelings at present, “cool” analysis of the future

We humans have a general tendency to be future oriented. There is an asymmetry when considering past and future events (Parfit, 1984). The pain I might have to endure tomorrow as part of a medical procedure is more disturbing than the pain I had yesterday. This bias makes me feel relieved that yesterday's pain is over, but makes me anxious about tomorrow's pain (and not the other way around). We are most concerned with what lies ahead of us. We are influenced by our personal views of the future – our expectations, fears, and hopes.

Besides the asymmetry between past and future, we are also strongly present oriented. Present feelings may actually be so powerful that considerations of future events are neglected. The term “temporal myopia” refers to the fact that whatever stands further away in time receives less consideration (Kim and Zauberman, 2009; Wittmann and Paulus, 2009). In Kurt Lewin's terms, we can say that the distant future is less present and affects a person's *life space* less or not at all: “The food that lies behind doors at the end of a maze so that neither smell nor sight can reach it is not a part of the life space of the animal. If the individual knows that food lies there this knowledge, of course, has to be represented in his life space, because this knowledge affects behavior.” (Lewin, 1951, p. 57).

**Fig. 1:**
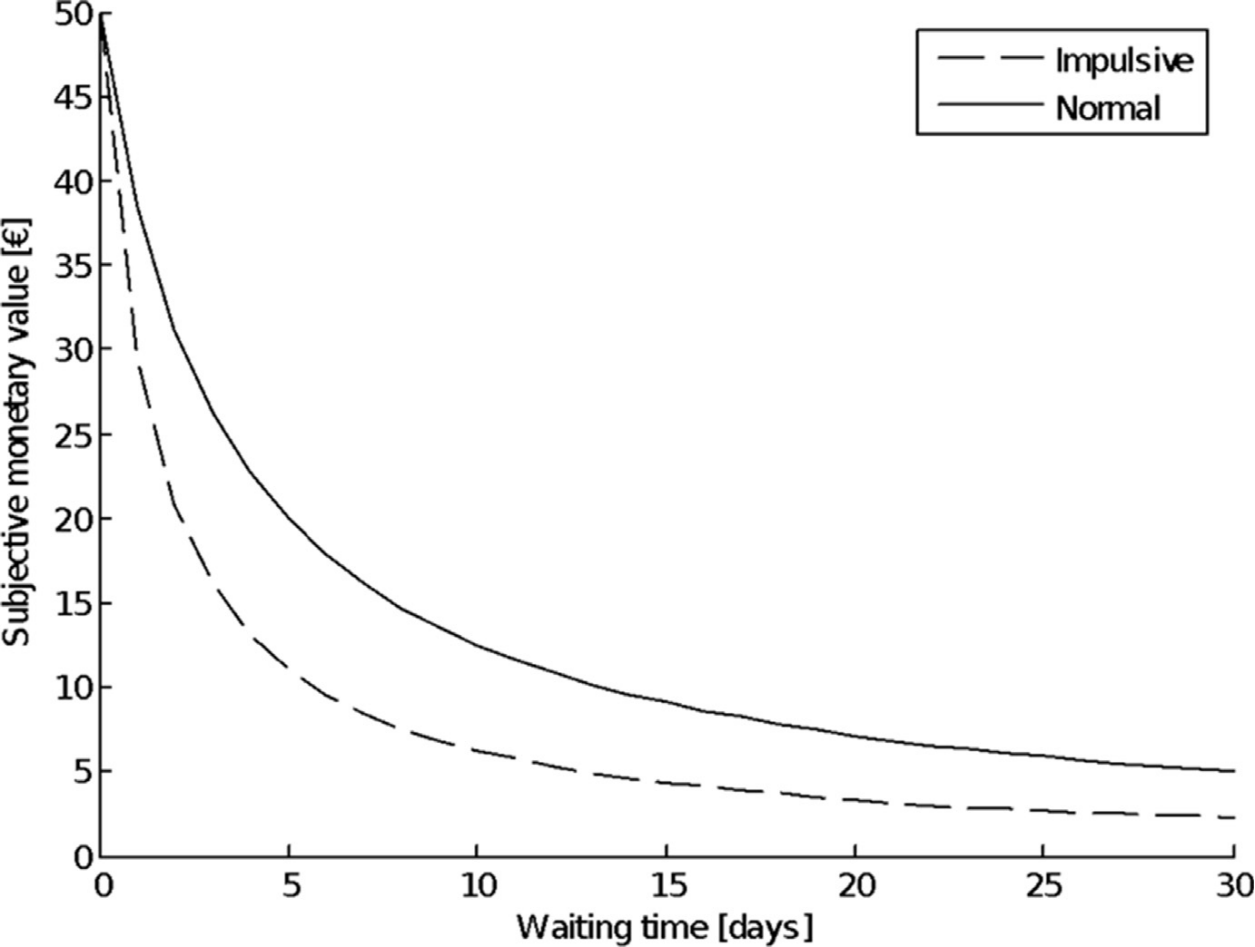
The hyperbolic curve represents the decrease in subjective monetary value of a reward when it is deferred (waiting time). As the waiting period increases, the subjective monetary value decreases, and the likelihood of choosing the delayed reward also decreases. For impulsive individuals, subjective monetary value is lower under all delay conditions.

Experimental studies clearly show that rewards that are received sooner are preferred over later rewards, as the subjective value of a utility is discounted as a function of the delay (Ainslie, 1975). This even holds true when the delayed reward is larger than the more immediate reward, e.g., people have to decide whether they want to receive €100 right away or €200 later. When the duration of the delay for the larger reward increases, preference for it decreases, and the likelihood of choosing the smaller reward increases. This temporal discounting pattern can be described by a hyperbolic function (Lane et al., 2003; Madden et al., 2003; Takahashi, 2009). This means that the value discounting function is steeper at shorter delay intervals (the rate of discounting is higher) and becomes flatter as the delay of the reward increases (the rate of discounting is lower) (see Fig. 1). Such value discounting functions similarly apply in intertemporal decision-making with regards to environmental and natural-resource policies. It has been shown that the value of the future benefits of an unpolluted natural environment decreases with increasing delay, i.e. regarding long-term issues, such as biodiversity or nuclear waste disposal. High up-front costs for mitigating the effects of the global climate change have to be spent now with century-long effects in a future that will not be experienced by today's decision makers (Carson and Roth Tran, 2009).

Related to the aforementioned stronger discounting of future goods by impulsive individuals, the discounting rate in those individuals is higher for all delays (Fig. 1), as has been shown in several studies with adolescents and adults (Baker et al., 2003; Barkley et al., 2001). Whereas impulsivity is considered to be a more or less stable personality trait, the experience of time varies considerably depending on fluctuating mental states in all people. In one study, participants who were sleep deprived transiently over-estimated duration and more strongly discounted future rewards (Reynolds and Schiffbauer, 2004). The future perspective was similarly modulated in another study during emotionally aroused states. Heterosexual male college students first had to rate photographs of sexually attractive female lingerie models or more neutral stimuli, such as rocks or trees (control group). In an immediately following session, subjects had to rate the subjective distance they felt to future time points. Participants who had previously rated the lingerie models anticipated future time to be farther away than those participants who had seen the less-exciting photographs (Kim and Zauberman, 2013). These findings can be interpreted that ‘heated’ affective states lead to a stronger focus on the present and ‘push’ the future farther away (Wittmann and Paulus, 2009, 2016).

In emotional situations, the ‘now’ is physically experienced in an ‘embodied’ way. ‘Later’ is beyond the horizon of immediate experience and, thus, hypothetical. That is what temporal construal theory is about (Trope and Liberman, 2003). Beyond a certain horizon of felt presence, temporally removed events tend to be assessed abstractly and analytically, not in emotional terms. Events occurring closer in time are evaluated more in the concrete dimension of affect that concerns us here and now. Whatever is experienced in temporal proximity elicits a greater range of physical and affective reactions, which influence decision-making. Due to this temporal proximity, a temporally closer option elicits interoceptive states which have greater impact – we are more likely to prefer immediate gratification (Loewenstein, 1996). The apparent possibility of reaching the desired goal increases the desire to reach it faster, immediately. Animals run faster as they approach their food box; prisoners show a disproportionate tendency to break out of jail shortly before they are due to be released (Papanek, 1974). The concreteness of mental representations for the present option accounts for typical delay-discounting behavior. The €100 now is real and ready for consumption; the €200 next week is a more abstract entity. Accordingly, many people tend to opt for the €100 now. If I am extremely hungry, I might not drive to my favorite restaurant with the highly-valued food, but pillage my fridge for immediate satisfaction. Similarly, and in relation to the example of an extreme drought, my urge to have a long, cool shower now is so emotionally dominant that the question of what will happen to the water supply over the next few days and weeks might not be taken into consideration.

A connection between the perception of time on the one hand and physical and affective processes on the other hand has actually been proposed in a model in the cognitive neurosciences (Craig, 2009, 2015; Wittmann, 2013, 2016). The experience of time would accordingly be related to emotional and visceral processes, as they share a common underlying neural system, the interoceptive system, including the insular cortex. Through an integration of bodily signals, processes converging to and diverging from the insular cortex are involved in creating the sense of an emotional moment. As a consequence, the sense of time would be created by successive moments of such self-realization, essentially formed by information originating within the body; felt emotions change the subjective passage of time and the judgement of duration. Emotions ‘here and now’ expand the felt presence. An emotionally aroused state leads to a relative overestimation of subjective duration and to the subjective delay of future events (Droit-Volet et al., 2013; Kim and Zauberman, 2013; Wittmann, 2016).

### 2.2 Personal and political life events: the dynamics of present and future orientation

An individual's time perspective can dynamically change according to situational demands on a short-term time scale. We can quickly and flexibly switch between time orientations, either focusing on the past to evaluate present options or imagining the future while creating alternative goals (Seginer and Lens, 2015). Decisions are therefore always based on the tripartite structure of temporal experience with a past, present, and future (Wittmann and Paulus, 2016). Although, as we will provide evidence below, temporal orientations are individually stable dimensions, to some extent they are malleable depending on the situational framing. When choices are framed in a way that subjects virtually “jump” in time towards a future option, and thus feel situationally closer, they relatively more often chose that option as compared to a framing of instructions where the option feels far away in the future, although objectively speaking there is no temporal difference between the options (Polunin, 2015). Through manipulations such as experimentally directing attention more to time in inter-temporal decision tasks time sensitivity is increased as earlier rewards are even more strongly preferred than later rewards (Ebert and Prelec, 2007).

In addition to such transient changes in time perception due to temporal cueing or emotional triggers, critical personal life events, i.e. unexpected unemployment or a life-threatening illness, can dramatically shorten a person's future perspective and lead to the dominance of the present perspective, as only short-term plans become relevant (Carstensen, 2006; van Laarhoven et al., 2011). This is a dominant feature of post-traumatic stress disorder, a strong present orientation at the expense of a future orientation (Goldrich, 1967; Lavi and Solomon, 2005; Misko and Tarabrina, 2004; Zimbardo et al., 2012). A traumatized person is less likely to believe that it is possible to control the future; future outcomes of present actions are not taken into consideration (Markus and Nurius, 1986).

Long-term societal crises also influence people's orientation in time (Nurkova and Vassilevskaya, 2003). Political crises are characterized by their novelty, i.e. there have been no similar situations in a person's experience, no point of reference in the past. Such a situation is characterized by unexpectedness and incoherence with existing values and the individual's worldview. These unexpected changes are viewed as a threat to experienced security and have a long-term impact on personal identity in relation to expectations, i.e. a person's future perspective is affected (Kobasa et al., 1982). The future becomes problematic, as it is no longer predictable, and the loss of certainty leads to goal disorientation and anxiety (Lewin, 1951; Marris, 1974; Salecl, 2004). Regarding the recent global financial crises, many people witnessed drastic changes in their usual ways of living, which affects time-related behavior. For example, in Australia a smaller percentage of people were considering retirement plans after the global financial crisis, which shows that the crisis influenced individuals' future planning and increased reliance on present activity (Griffin et al., 2010). In the late 1990s, Muzdybaev (2000) carried out extensive research in Russia on the period after the collapse of the Soviet Union and the Russian financial crisis in 1998, when inflation reached 84 percent as compared to the year before, welfare costs increased considerably, and many banks closed. As a consequence, the time span people considered for planning for the future narrowed down. People experienced temporal disorientation and became more strongly orientated towards the past. Another study on changes in time perspectives after the financial crisis of 2008/2009 in Russia showed that people became less future oriented, and the present was viewed more hedonistically and less fatalistically. This latter change was interpreted as a coping strategy in the face of an unpredictable future (Zarubin and Sircova, 2013). In times of political and economic turbulence, the future orientation recedes, and the present orientation takes precedence.

### 2.3 Time in perspective: personality influences present and future orientation

When people show a relative dominance of one time orientation, it can be considered a personality trait. For example, impulsive behavior is defined as generally reacting to the immediate situation without thinking about future consequences. In this way, impulsivity is conceptualized as extreme temporal short-sightedness or as dominance of the present orientation. In Zimbardo and Boyd's concept of time perspective (TP) as related to their self-rating questionnaire, in the Zimbardo Time Perspective Inventory (ZTPI; Zimbardo and Boyd, 1999; see Glossary), the subscales of the present hedonistic and present fatalistic orientation are correlated with trait impulsivity (Baumann and Odum, 2012; Lee and Song, 2011; MacKillop et al., 2006; Sircova et al., 2008). Behaviorally, a stronger focus on the present at the expense of the future is associated with the propensity to take risks (Jochemczyk et al., 2017) and is related to impulsive behavior, such as gambling, having unprotected sex, and using drugs (Keough et al., 1999; Zimbardo et al., 1997). People who are more future-oriented exhibit greater moral concerns compared to more present-oriented people (Agerström and Björklund, 2013). This is explained by the above-mentioned construal-level theory (Trope and Liberman, 2003). People who are more future-oriented are able to analyze morally relevant behavior at a higher level of abstraction and, therefore, foresee potential future consequences in greater detail (Agerström and Björklund, 2013). Accordingly, future-oriented individuals are more prone to display pro-environmental behavior. As has been shown in a meta-analysis with 19 independent studies in seven countries spread over the world, future orientation is actually a better predictor of pro-environmental behavior than having ‘green’ attitudes (Milfont et al., 2012). These are of course correlational findings and the explained variance of behavior is low to moderate. Moreover, recent empirical research is indicative of the domain specificity of scores regarding future orientation (McKay et al., 2017; McKay et al., 2018); i.e. someone may be future oriented regarding sustainability issues but not when she is concerned with academic success or a retirement plan. Nevertheless, when looking at moderate correlation coefficients between scores, individuals who are more future-oriented show more considerate and reasonable behavior, and they are more conscious of the environment, whereas individuals with a dominant present orientation are more likely to engage in more risky and unhealthy behaviors, such as risky driving, alcohol and substance abuse, fewer HIV-preventive behaviors, etc (Boyd and Zimbardo, 2005). In employing the two-factor model of consideration of future consequences (CFC; Joireman et al., 2008), it has been shown that pro-environmental motivation is mainly driven by a reduction in immediate concerns (Arnocky et al., 2014). Future TP has also been shown to be associated with achievement and success at school and university – students with a more pronounced future-oriented TP show more goal-oriented behavior and are engaged in a greater number of activities related to achieving these goals (Boyd and Zimbardo, 2005).

One must differentiate between an impulsive present orientation and present-mindedness as developed and cultivated, for example, through introspective training, such as meditation (Campos et al., 2016; Schötz et al., 2016; Wittmann and Schmidt, 2014). The former is associated with a strong urge to act at the present moment and is stimulus-oriented, whereas the latter is associated with an observational state related to more self-control (Short et al., 2016; Wittmann et al., 2014). One would have to conceptualize impulsivity as an urge to act in the immediate future versus acting in the interest of long-term goals (Joireman et al., 2012). Highly-impulsive individuals are focused on or “pulled” into a very near future in which they will be rewarded with distraction and positive reinforcement. Therefore, one also has to consider a positive present orientation. A positive focus on the present is relevant and instrumental for human functioning and well-being (Bajaj and Pande, 2016; Nezlek et al., 2016). In one recent concept of integrating ‘mindfulness’ and ‘flow’ as mental constituents of a positive present orientation, an inventory assessing the present-eudaimonic time orientation was developed (Vowinckel et al., 2017; the Greek *eudaimonia* means happiness or ‘human flourishing’). ‘Mindfulness’ was taken as a mode of consciousness characterized by an open, non-judgmental awareness of what is happening at the present moment (see below for a more detailed account), and ‘flow’ is defined as the state of being happily absorbed when experiencing and functioning optimally (Csikszentmihalyi and Csikszentmihalyi, 1992).

The processes of weighing options relating to past, present, and future all take place in an extended present; switching the time perspective adequately may be bound to a mindfully-oriented present (Vowinckel et al., 2017). ‘Mindfulness’ is a concept taken from Buddhist psychology and is understood as bringing awareness to each present moment with an accepting and non-judgmental attitude (Kabat-Zinn, 2005). Being mindful is related to increased emotional self-control, i.e. the capacity for inhibitory control over inner impulses and immediate urges (Sauer et al., 2011; Sauer et al., 2012; Wittmann et al., 2014). Being mindfully present means that one is aware of what is going on right now without shying away from negative feelings. It is the opposite of a habitual mode with automatic reactions. Such meta-awareness of one's mental life is thus related to more self-control, which is important when deciding between tempting short-term behaviors and more personally costly sustainable behaviors (Ericson et al., 2014). Since we humans have the tendency to choose those options that come most easily and automatically to mind (system 1), as opposed to choosing options that are realized after effortful and cognitively highly-controlled activity (system 2), being mindful can also be seen within the dual-process theory as enhancing effortful, more sustainable decision-making (Kahneman, 2011).

Within the context of eco-psychology, a mindfully-oriented present awareness can also be seen in another, complementary way as connecting the self inextricably with nature (Amel et al., 2009). In essence, if you feel you are part of nature, you don't litter it. Sustainable behavior naturally evolves from an attitude of caring for nature as a form of self-care. This idea fits the notion of non-duality – non-separation between nature and self as developed in spiritual practice. What is formulated in these terms of mindfulness practice can also be expressed in a more profane way in systems biology. As an organism within the ecosystem of this planet, whenever I pollute air, water, and soil, I pollute the air I breathe, the water I drink, and the food I eat. As empirical work shows, an increased connectedness to nature really is associated with trait-mindfulness and everyday pro-environmental behavior (Barbaro and Pickett, 2016). Empathy and compassion are also essential components of inter-individual cooperation, traits that can be developed individually and in societies (Nussbaum, 2003). Being mindful of others' needs, as well as a non-materialistic attitude, has been shown to be predictive of sustainable behavior as well (Ericson et al., 2014).

### 2.4 Glossary

The **Zimbardo Time Perspective Inventory** (**ZTPI**; Zimbardo and Boyd, 1999) contains 56 five-point items ranging from 1 (very untrue) to 5 (very true). An individual reports his or her time orientation in one of the five subscales below:

(1) **Past negative**: “I often think about the bad things that have happened to me in the past”
(2) **Past positive**: “Happy memories of good times spring readily to mind”
(3) **Present hedonistic**: “I take risks to put excitement in my life”
(4) **Present fatalistic**: “Because things always change, one cannot foresee the future”
(5) **Future**: “I am able to resist temptations when I know that there is work to be done”

It can be argued that these dimensions are not sufficient to capture all aspects of human time perspective. A negative future dimension and a positive present dimension are missing. Therefore, a Swedish version of the ZTPI that has a six-factor structure comprising a **future negative** scale was developed and validated: “To think about my future makes me sad” (Carelli et al., 2011). Several scales assessing a form of **mindful present orientation** exist (Sauer et al., 2013). The **Freiburg Mindfulness Inventory** (FMI; Walach et al., 2006) has a two-factor structure with the subscale of “presence”, which is defined as an ability to attend to the present moment: “I am open to the experience of the present moment.” Another form of present orientation was recently conceptualized and a corresponding questionnaire developed: the **Present-Eudaimonic** scale, which encompasses two present-directed concepts, mindfulness and flow (Vowinckel et al., 2017).

An ideal profile of a **balanced time perspective** (**balanced TP**) can be derived from the five dimensions of the ZTPI, which contains high scores on the past-positive time perspective, moderately high scores on the future and the present-hedonistic time perspective, and low scores on the past-negative and the present-fatalistic time perspective (Zimbardo and Boyd, 2010). In this approach the **deviation from the balanced time perspective** (**DBTP**) is as measure of how much an individual's empirical values across the five dimensions differ from this ideal value (Stolarski et al., 2015).

One further conceptually-based approach was developed by Wiberg et al. (2012), where a person with a **balanced TP** profile would score in the following way on items ranging from 1 (low score) to 5 (high score):

- Low on the past-negative, present-fatalistic, and future-negative scales. Individuals should score 1 or 2 on most items (for the exact scoring, see Wiberg et al., 2012).
- Moderate on the present-hedonistic scale. Individuals should score 3 on most items.
- Moderately high on the future and future-positive scales. Individuals should score 3 or 4 on most items.
- High on the past-positive scale. Individuals should score a 4 or a 5 on most items.

A person's balance level depends on how many TP dimensions are in the balanced range. For example, a person with the level 5 has a fully balanced TP, as s/he scores in the desired range on all five dimensions.

### 2.5 A mindfully balanced time perspective

The dominance of a single time orientation cannot be considered optimal for human functioning and well-being. An overly strong focus on the future can potentially impair quality of life. Having an emotionally rewarding existence depends on the hedonistic capacity to live for the moment, such as spontaneously agreeing to spend an evening with friends. Regret about exercising self-restraint, i.e. when everybody else went to a great party, actually proves much stronger than regret about giving in to temptation (Kivetz and Keinan, 2006). Whether one lives at the spur of the moment or pursues long-term goals is a matter of emotionally intelligent conduct of weighing options in life and having a balanced time perspective (Boniwell and Zimbardo, 2003; Stolarski et al., 2011; Wittmann, 2016). Referring specifically to the five dimensions of the ZTPI (Zimbardo and Boyd, 1999; see the Glossary), one could define a balanced time perspective (balanced TP; see the Glossary) as scoring moderately high on the future and the present-hedonistic dimension and with high scores on the past-positive dimension. A balanced TP is also related to low scores on the past-negative and on the present-fatalistic dimensions (Boniwell and Zimbardo, 2004; Stolarski et al., 2015; Zimbardo and Boyd, 2010) and is negatively associated with the future-negative dimension (Wiberg et al., 2012). In essence, someone with a balanced time perspective has the propensity to switch flexibly among the time orientations of past, present, and future.

It has been demonstrated that people with a balanced TP are happier (Boniwell et al., 2010), effectively cope with stressful life situations (Sircova and Mitina, 2008), and are more satisfied with their lives (Boniwell et al., 2010; Sircova and Mitina, 2008), even after controlling for influences of other basic personality traits (Stolarski, 2016). Individuals with a balanced time perspective have beliefs and a sense of directedness that give life purpose (Boniwell et al., 2010; Sircova and Mitina, 2008); they are also relatively more optimistic and confident in their abilities to achieve goals (Boniwell et al., 2010), and they feel less time pressure, as the passage of time in their lives is experienced to pass more slowly (Wittmann et al., 2015). People who have this profile are conscious of living in the present, i.e. in the sense of a mindfulness present orientation, and are able to integrate the present, past, and future orientations into a coherent whole (Wiberg et al., 2012; Wiberg et al., 2017). Moderate correlation coefficients indicate that a balanced TP is a form of mindful present orientation (Stolarski et al., 2016). Mindfulness might actually be the most important component of the balanced time perspective. Seema and Sircova (2013) showed how self-rated mindfulness explained the largest variance in the balanced TP among all other individual time orientations of the ZTPI. The authors argue that mindfulness is both a time perspective and is also related to meta-awareness of one's own time perspective.

The psychological, behavioral, and attitudinal profiles of seven individuals with a balanced TP (as assessed with the ZTPI in a group of 50 people) were explored in a recent investigation with interviews (Wiberg et al., 2017). According to the ZTPI scores, people with a balanced TP, compared to people without this balance, had a more positive attitude and gratitude towards one's own past, which becomes a foundation for a strong feeling of self-esteem and a sense of self-efficacy. At the same time, they strongly anticipate future-oriented activities; they project themselves into the future and make plans over the course of the coming years. People with a balanced TP enjoy activity per se and do not only focus on the product of their efforts, as would a solely future-oriented person. When necessary, they can resist temptations at the present moment. During the interviews it also became apparent that individuals with a balanced TP are aware of death and accept their own mortality. They can also think of what will happen to others and the environment when they are gone; they display a range of sustainable attitudes, as well as behaviors, like sorting garbage and being vegetarian because of the wish to reduce meat production. Although they are aware that these behaviors will only make a small difference, they believe that it is important to act in this way (Wiberg et al., 2017).

## 3 Conclusions

Policy making regarding sustainable development has to address a mixture of temporal and social dilemmas (Milfont et al., 2012; Sircova et al., 2015). Sustainable development is based on long-term goals: it requires one to adopt anticipatory behaviors, to imagine the future, and consider the consequences of one's own behavior. Since this long-term outlook may conflict with short-term interests, policy making addressing sustainability issues is a form of intertemporal decision making, essentially a trade-off between current and future benefits. Addressing environmental issues also involves the management of limited resources. Here the social dilemma comes into play, as an individual might have to decide between personal comfort and profit, and the abstract concept of collective interest.

In an ethical sense, our generation ought to adopt behavioral styles that would slow down or even counter negative environmental effects, i.e. climate change. Policy making happens within the context of psychological time. Due to the long delays of certain effects from pro-environmental decision making, today's ecologically-minded behavior will only benefit future generations. The effects will not be experienced by those politicians who implement the measures. Personal temporal conflicts could, therefore, be paraphrased as “Why should I restrain my comfort because it would benefit people I will never meet? I want to have a good life now”. Such an attitude is not expressed openly and publicly. Dissonance reduction – not wanting to be seen as an egocentric person – may be one reason for the denial of climate change: “If there is no climate change that affects generations to come, then I don't have to constrain myself.”

The role of individual differences in the experience of time is clearly a major factor in explaining different attitudes and behaviors fostering sustainable development. Humans in general prefer sooner over later rewards. Positive consequences that lie too far into the future are discounted so that outcomes with shorter delays are preferred. We presented and discussed different temporal-orientation patterns in individuals and how they relate to pro-environmental behavior. A strong hedonistic present orientation at the expense of future considerations is a negative predictor of sustainable behavior; a strong future orientation and a mindful present orientation are positive predictors of sustainable behavior. We discussed how a balanced time perspective as mindful meta-awareness of time orientation is related to both personal and collective well-being. Those individuals who have a balanced time perspective feel more connected to the overall developments that evolve in the world. They appreciate what the past has provided, they are more aware of the present moment, feel connected to nature, and are more conscious of their everyday actions and decisions and how those influence the future.

We conclude that a balanced time perspective is an individual trait one could strive to foster in everyone. This could be a goal for education programs starting from kindergarten and including life-long learning at all ages. We have seen that massive societal changes can abruptly change the time perspective of an individual. Independent of decades-long debates regarding the controversial nature-nurture question on personality development, it is fair to say that there is always room for learning and for behavioral changes. Empirical studies are indeed witness to such changes in which children were made more future-oriented through mentally visualizing future goals (Zimbardo and Boyd, 2010). In order to save the eco-system of the planet it would be helpful if more people developed a balanced time perspective.

Our overview is an attempt to bring together concepts from time psychology and eco-psychology. First of all, we embedded these findings not only within the psychology of time perspective but also within a broader approach based on cognitive psychology and behavioral economics. Then we summarized converging evidence on the predictive power of individual differences in time perspective, notably regarding the future orientation and the mindful present orientation. Moreover, this overview is novel as it integrates the concepts of mindfulness and, especially, balanced time perspective for understanding issues in eco-psychology. These concepts overall have entered psychological theorizing only recently. However, a systematic empirical study assessing the balanced time perspective in relation to various outcome measures in eco-psychology has yet to be conducted. Studies will have to broaden the empirical basis of our claims but they will also have to distinguish underlying predictors or confounding concepts on individual differences regarding the terms ‘mindfulness’ and ‘balanced time perspective’. For example, future research will disclose the underlying neurocognitive (working memory, inhibition, task switching) and emotional processes at play that relate to concepts of temporal metacognition and self-control and how they predict sustainable behavior in individuals having a mindful and balanced time perspective (Esch, 2014; Stolarski and Witowska, 2017). In our overview we focused on time related individual differences. Another concept is also of importance: systems thinking as a cognitive capacity to understand the behavior of complex adaptive systems (Davis and Stroink, 2016). Empirical work has shown that a heightened capacity to think in systems, i.e. to realize that a dynamic whole is made up of interconnected components, is related to ascribing more value to the natural world and to showing more engagement for pro-environmental behavior (Davis and Stroink, 2016; Lezak and Thibodeau, 2016). To assess the intersection between ‘systems thinking’ and the discussed time orientations is a further line of future research.

A final, critical, note has to be added. The non-linear complex dynamics of the ecological system makes it difficult, not only for lay person but also for experts, to identify what pro-environmental behavior is (Thibodeau et al., 2016). For example, as has been argued, the different environmental and health-related effects organic and conventional farming have is much more complex than a simple ideological dichotomy of “good” and “bad” would imply (Trewavas, 2001). Another example is that hybrid and electric cars have an environmentally advantageous effect only if the used electricity is taken from renewable energy sources (Granovskii et al., 2006). That said, certain behavior options can be easily judged by its transparent nature such as the decision between taking the car to work or walk.

When we postulate an influence of time orientation on environmental decision-making we claim that these trait-like perspectives explain only a certain amount of variance in behavior. Naturally, there are multiple factors at work. Over the whole population, monetary constraints such as a rise in fuel prices are a more potent cause for monetary investments in energy saving technology than green attitudes (Black et al., 1985). Opting for pro-environmental behavior is more likely when presumed benefits are directly apparent (but, as we saw above, need not be actual benefits) and the inconvenience and costs are not too high (Turaga et al., 2010). On the other hand, as has been empirically shown and conceptually developed, smaller communities who are left on their own without top-down administrative intervention can develop a form of “Governing the Commons”. This means that when individuals in small-scale communities actually communicate with each other they do not necessarily deplete the resources and act in a sustainable way (Ostrom, 2010).

Many problems however have to be solved on the larger scale of cities, states, and the whole world. The related governmental units have to decide on many larger-scale issues where people cannot anymore communicate directly. These decisions are made by individual politicians and they have to be backed by the people. People however are different. The variations in values, beliefs, and attitudes make up differences in character that varies between selfish hedonists and altruist who stand for the common good (Sircova et al., 2015; Turaga et al., 2010). Some of the variation in these personality factors can be mapped onto an individual combination in the expression of stable time orientations (Sircova et al., 2015). What we wanted to show is that the time perspective is one factor (among many) explaining behavior and attitudes related to the natural environment. We hope that with our overview we help to pave a theoretical path for future empirical studies.

## Declarations

### Author contribution statement

All authors listed have significantly contributed to the development and the writing of this article.

### Funding statement

This research did not receive any specific grant from funding agencies in the public, commercial, or not-for-profit sectors.

### Competing interest statement

The authors declare no conflict of interest.

### Additional information

No additional information is available for this paper.
